# Human 3D nucleus pulposus microtissue model to evaluate the potential of pre-conditioned nasal chondrocytes for the repair of degenerated intervertebral disc

**DOI:** 10.3389/fbioe.2023.1119009

**Published:** 2023-02-14

**Authors:** Jesil Kasamkattil, Anna Gryadunova, Raphael Schmid, Max Hans Peter Gay-Dujak, Boris Dasen, Morgane Hilpert, Karoliina Pelttari, Ivan Martin, Stefan Schären, Andrea Barbero, Olga Krupkova, Arne Mehrkens

**Affiliations:** ^1^ Spine Surgery, University Hospital Basel, Basel, Switzerland; ^2^ Department of Biomedicine, University of Basel and University Hospital Basel, Basel, Switzerland; ^3^ World-Class Research Center “Digital Biodesign and Personalized Healthcare”, Sechenov First Moscow State Medical University, Moscow, Russia; ^4^ Department of Biomedicine, Institute of Anatomy, University of Basel and University Hospital Basel, Basel, Switzerland

**Keywords:** nucleus pulposus, degenerative disc disease, cell therapies, spheroids, chondrospheres, IL-1Ra, 3D *in vitro* models

## Abstract

**Introduction:** An *in vitro* model that appropriately recapitulates the degenerative disc disease (DDD) microenvironment is needed to explore clinically relevant cell-based therapeutic strategies for early-stage degenerative disc disease. We developed an advanced 3D nucleus pulposus (NP) microtissues (µT) model generated with cells isolated from human degenerating NP tissue (Pfirrmann grade: 2–3), which were exposed to hypoxia, low glucose, acidity and low-grade inflammation. This model was then used to test the performance of nasal chondrocytes (NC) suspension or spheroids (NCS) after pre-conditioning with drugs known to exert anti-inflammatory or anabolic activities.

**Methods:** NPµTs were formed by i) spheroids generated with NP cells (NPS) alone or in combination with ii) NCS or iii) NC suspension and cultured in healthy or degenerative disc disease condition. Anti-inflammatory and anabolic drugs (amiloride, celecoxib, metformin, IL-1Ra, GDF-5) were used for pre-conditioning of NC/NCS. The effects of pre-conditioning were tested in 2D, 3D, and degenerative NPµT model. Histological, biochemical, and gene expression analysis were performed to assess matrix content (glycosaminoglycans, type I and II collagen), production and release of inflammatory/catabolic factors (IL-6, IL-8, MMP-3, MMP-13) and cell viability (cleaved caspase 3).

**Results:** The degenerative NPµT contained less glycosaminoglycans, collagens, and released higher levels of IL-8 compared to the healthy NPµT. In the degenerative NPµT, NCS performed superior compared to NC cell suspension but still showed lower viability. Among the different compounds tested, only IL-1Ra pre-conditioning inhibited the expression of inflammatory/catabolic mediators and promoted glycosaminoglycan accumulation in NC/NCS in DDD microenvironment. In degenerative NPµT model, preconditioning of NCS with IL-1Ra also provided superior anti-inflammatory/catabolic activity compared to non-preconditioned NCS.

**Conclusion:** The degenerative NPµT model is suitable to study the responses of therapeutic cells to microenvironment mimicking early-stage degenerative disc disease. In particular, we showed that NC in spheroidal organization as compared to NC cell suspension exhibited superior regenerative performance and that IL-1Ra pre-conditioning of NCS could further improve their ability to counteract inflammation/catabolism and support new matrix production within harsh degenerative disc disease microenvironment. Studies in an orthotopic *in vivo* model are necessary to assess the clinical relevance of our findings in the context of IVD repair.

## 1 Introduction

Low back pain (LBP) is experienced by 80% of the world population at least once in their life and it is one of the costliest diseases for the healthcare system (Frapin et al., 2019). 40% of the chronic LBP cases are due to degeneration of the intervertebral disc (IVD) (Schwarzer et al., 1995; Luoma et al., 2000). During IVD degeneration, the extracellular matrix (ECM) of the nucleus pulposus (NP) tissue, especially the proteoglycans (PG), is degraded due to the imbalance of catabolic and anabolic activities (Struglics and Hansson, 2012; Dowdell et al., 2017) and the cell density in the IVD decreases over time (Zhao et al., 2007). The gradual onset of IVD degeneration is considered as a part of the natural course of ageing (Sakai and Andersson, 2015). However, with progressing degeneration, the NP tissue could become inflamed and/or herniate through annulus fibrosus (AF) and press against the nerve roots causing pain (Nakazawa et al., 2018). The association of IVD degeneration with inflammation and pain is referred to as degenerative disc disease (DDD). Surgical strategies to treat DDD often do not improve patient’s quality of life, as they could accelerate degeneration of adjacent IVDs (van den Eerenbeemt et al., 2010; Deyo and Mirza, 2006). In order to prevent surgery, minimally invasive biological therapies should be developed and applied at relatively early stage (Frapin et al., 2019). Ideally, these will restore the structure and function of the mildly affected NP (Pfirrman grade 2–3), allowing the IVD to hydrate and regain the height, as well as reduce the catabolic shift (Binch et al., 2021). However, despite the advancement in biological IVD repair (Binch et al., 2021), no therapy has been widely adopted clinically yet (Meisel et al., 2019; Urits et al., 2019; Eisenstein et al., 2020).

Numerous *in vitro*, *ex vivo*, and *in vivo* studies have investigated the effects of anti-catabolic or anti-inflammatory factors on IVD repair (Seki et al., 2009; Walter et al., 2015; Evashwick-Rogler et al., 2018). The aim was to either directly suppress the expression of catabolic enzymes i.e., matrix metalloproteases (MMPs) and a disintegrin and metalloproteinase with thrombospondin motifs (ADAMTS) (Klawitter et al., 2012; Kim et al., 2013) or by downregulating pro-inflammatory mediators (i.e., tumor necrosis factor alpha (TNF-α), interleukin-1 (IL-1) (Le Maitre et al., 2007a; Sinclair et al., 2011). Although these anti-catabolic and anti-inflammatory therapeutic strategies are promising, they are not sufficient enough to regenerate the IVD function since the resident cells often fail to restore their ability to synthesize ECM (Frapin et al., 2019). Pro-anabolic strategies using growth factors i.e., transforming growth factor β (TGFβ) or growth differentiation factor 5 (GDF-5) have been also investigated to induce matrix formation within the NP tissue with encouraging *in vitro*, *ex vivo* and *in vivo* results (Thompson et al., 1991; Nishida et al., 1998; Li et al., 2004; Chujo et al., 2006; Liang et al., 2010). As an example, clinical studies evaluated the safety, tolerability and efficacy of GDF-5 injection into degenerating IVD, with no major adverse events directly related to GDF-5 injection as well as moderate improvement of pain and disability (https://clinicaltrials.gov; NCT01158924, NCT00813813, NCT01182337, and NCT01124006). However, these pro-anabolic approaches are hampered by the limited amounts of healthy/metabolically active cells in the degenerated NP. Therefore, a single intradiscal injection of biological factors with anti-catabolic, anti-inflammatory and pro-anabolic effects combined with healthy therapeutic cells, which survive and produce ECM within the DDD microenvironment, could be a better approach to repair the NP tissue. Nevertheless, translation of cell-based approaches for IVD repair still faces several critical challenges, mainly related to (i) selection of a therapeutic cell source with good performance within the DDD microenvironment and (ii) a lack of an *in vitro* model that appropriately mimics the course of the disease and at the same time allows for clinically relevant incorporation of therapeutic cells (Buckley et al., 2018; Smith et al., 2018; Thorpe et al., 2018).

To repopulate the NP tissue with cells, endogenous stem/progenitor cell recruitment by injecting chemokine ligands (i.e., chemokine (C-C motif) ligand 5 (CCL5) or C-X-C motif chemokine 12 (CXCL12)) (Gruber et al., 2014; Frapin et al., 2020; Zhou et al., 2020) or exogenous cell injection strategies have been explored over the last two decades (Frapin et al., 2019). For exogenous cell injection, differentiated cell sources (NP, AF, articular chondrocytes (AC)) as well as stem/stromal cells (derived e.g. from bone marrow or adipose tissue) in combination with or without scaffolds were investigated (Williams et al., 2021; Kasamkattil et al., 2022). However, for both endogenous and exogenous cell supplementation, healthy cells are either not available in sufficient number, donor site morbidity arises, and/or cell survival within the harsh DDD microenvironment is reduced (Sakaguchi et al., 2005; Wuertz et al., 2009; Vadala et al., 2012; Sakai and Andersson, 2015; Vadala et al., 2019). These limitations can be overcome by using nasal chondrocytes (NC), isolated from autologous nasal septum cartilage with minimal donor site morbidity (Siegel et al., 2000; Homicz et al., 2002; Fulco et al., 2014). NC show superior viability over AC and mesenchymal stromal cells (MSCs) in simulated DDD microenvironment, thus representing a robust cell population with a likelihood of survival post injection (Gay et al., 2019). We have demonstrated that spheroids formed with NC (hereafter referred to as nasal chondrocyte spheroids, NCS) generate own matrix, and survive and fuse with NP microtissues in DDD microenvironment (Gryadunova et al., 2021). Notably, NCS are injectable into the IVD using a spinal needle, without losing their structural integrity (Gryadunova et al., 2021). Therefore, NCS represent a promising alternative for single-injection-based IVD repair strategy.

The first step towards developing a functional cell-based strategy for IVD repair includes testing in *in vit*ro models. At this stage, general proof of principle, intercellular communications, cell functions, and cell behavior are investigated. For the appropriate design of the *in vitro* models, the selection of ideal cell source, culture system and culture condition are of key importance. Also, the right choice of species from which resident/therapeutic cells are being isolated for *in vitro* culture has to be considered because it is known that species-specific responses could lead to different outcomes (Bach et al., 2017; Rosenzweig et al., 2017).

Interaction between IVD and therapeutic cells is commonly investigated in 2D and 3D co-culture models, tissue explants as well as organ culture models (Thorpe et al., 2018). 3D culture models restore the IVD cell phenotype and allow to reproduce *in vivo* spatial distribution of the IVD cells, which makes them more physiologically relevant and predictive than 2D monolayer cultures (Ravi et al., 2015). Different 3D *in vitro* co-culture models have been used to study the interaction between therapeutic and IVD cells (Choi et al., 2011; Naqvi and Buckley, 2015; Li et al., 2019). As an example, direct co-culture of human bone marrow stromal cells (BMSCs) with bovine NP cells (ratio: 1:1) encapsulated in 3D alginate beads revealed that hypoxia and co-culture could lead to BMSCs differentiation into NP-like phenotype (Stoyanov et al., 2011). In order to overcome several disadvantages of the alginate bead model, such as lack of reproducibility and uniformity of quality and size of the microspheres (Lee et al., 2001), the 3D pellet culture model could be used (Vadala et al., 2008; Svanvik et al., 2010; Watts et al., 2013). However, even the direct co-culture pellet model does not simulate the *in vivo* situation properly since therapeutic cells are not supposed to differentiate with differentiating NP cells but should rather be introduced to an already differentiated NP microtissue. Tissue explants and organ culture models are excellent to test local tissue responses, integration and delivery of therapeutic cells into IVD tissue and also in regard to biological and cellular functions (Zhang et al., 2008; Le Maitre et al., 2009a; van Dijk et al., 2014; van Dijk et al., 2017). However, due to the handling and complexity of tissue/organ culture, they are not well suited for more fundamental cellular mechanistic studies.

Selecting the ideal culture condition to mimic the DDD microenvironment is essential to study the potential of therapeutic cell for IVD regeneration. The harsh NP microenvironment is characterized by avascularity, hypoxia, low glucose level, acidity, inflammation, high osmolality and restricted biomechanics (Dou et al., 2021). Several studies have assessed the performance of the therapeutic cells within *in vitro* models simulating some of the parameters present in the DDD microenvironment (Vadala et al., 2019). Nevertheless, it has been shown that less than 15% of the *in vitro* studies include solely of these parameters thus not mimic the harsh IVD microenvironment properly (Thorpe et al., 2018).

**FIGURE 1:**
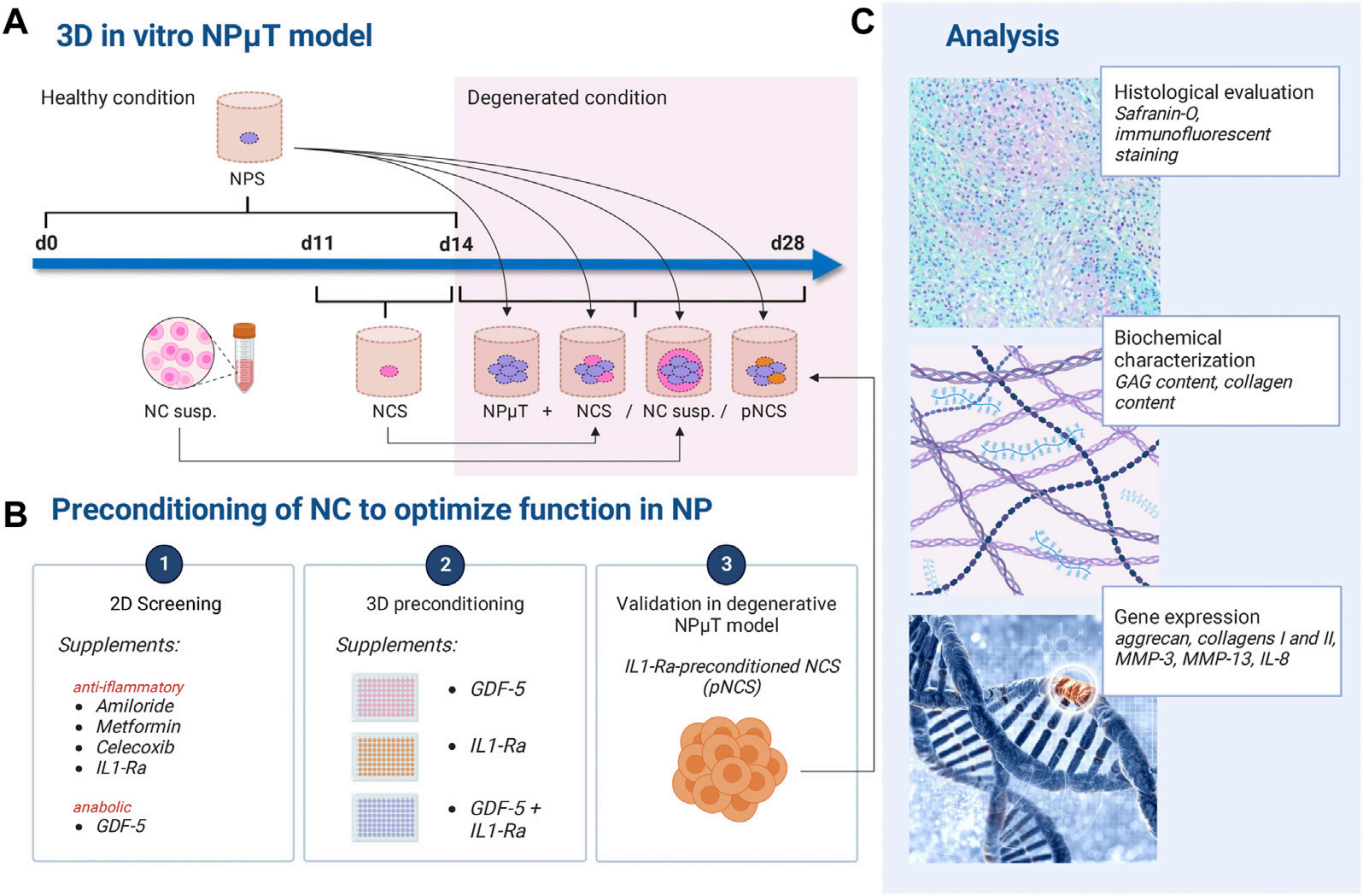
Experimental design. **(A)** NP microtissue (NPμT) model: single spheroids were generated by culturing NC (NCS) and NP cells (NPS) for 3 days or 2 weeks respectively, in healthy condition. Afterwards, NPS were pooled with NCS/NC to form NPµT (+ NCS/NC susp). **(B)** NC were preconditioned with anti-inflammatory or pro-anabolic compounds in **(B1)** 2D culture and **(B2)** 3D culture. **(B3)** IL-1Ra-preconditioned NCS (pNCS) were implemented in the NPµT model. **(C)** Histological, biochemical, and gene expression analyses were performed. Created with BioRender.com.

Since available 3D models are still not satisfactory to study the responses of therapeutic cells to the harsh NP microenvironment, we aim to develop a practical yet sufficiently complex 3D NP microtissues (µT) model. Within this NPµT model we investigate the responses of therapeutic cell suspensions as well as cell spheroids (NC vs. NCS) to the DDD microenvironment. Furthermore, we test the efficacy of different clinically relevant compounds to improve NC function once exposed to DDD microenvironment (Figure 1). We demonstrate that the model is suitable to test pre-conditioning strategies that enhance the NP repair potential of therapeutic cells.

## 2 Materials and methods

### 2.1 Cells and cell sources

#### 2.1.1 Tissue harvest and cell isolation

Tissues were collected following local ethical committee approval (EKNZ-2015305, University Hospital Basel). After obtaining informed consent from all donors, human nasal septal cartilage tissue was harvested from the patients undergoing rhinoplasty (total *n* = 6, Supplmentary Table S1). NP tissue was acquired from donors undergoing surgery for DDD. Harvested NP tissues were graded using Pfirrmann scale (Urrutia et al., 2016). NP tissues with Pfirrmann grade 2–3 (mild/moderate degeneration) were used in this study (total *n* = 6, Supplementary Table S2). NC and NP cells were isolated after digestion in collagenase type II (0.15% for NC; 0.05% for NP cells for 22 h), and expanded in NC and NP expansion medium respectively (composition in Supplementary Material) up to passage 3.

#### 2.1.2 Lentiviral transduction of nasal chondrocytes

In order to distinguish NC from NP cells, NC were labelled with mEmerald fluorescent protein using lentiviral transduction. Lentiviral expression vector was generated by subcloning mEmerald coding sequence by PCR from pmEmerald-LifeAct-7 vector (Addgene, 54148) into pLVX lentiviral vector (Clontech, United States). For lentivirus production, Lenti-X 293T cells (Clontech, United States) were transfected with lentiviral expression vector and 3rd generation packaging plasmids prMDLg/pREE, pRSV-Rec, and pMD2.G (Addgene, 12251, 12253, and 12259, respectively) using Lipofectamine 2000 (Lifetech, 11668019). After 72 h, the supernatant containing lentiviral particles was collected, and lentiviral titer was assessed by ELISA using Quick Titer Lentivirus titer kit (Cell Biolabs, VPK-1070). Lentiviral transduction of NC was performed according to previously established protocol, reported affecting neither proliferation nor the differentiation capacity of chondrocytes (Miot et al., 2010). Briefly, NC were seeded in 6-well plates as 2.5 × 10^6^ cells/well and transduced with mEmerald-lentivirus at MOI 5 in the presence of 8 µg/ml polybrene, which yields ≥95% transduction efficiency (TEf). TEf was monitored by yielded the percentage of green-positive cells by flow cytometry (Aria III, BD) 3 days post-transduction.

### 2.2 Drug screening in 2D culture of nasal chondrocytes

NC (*n* = 3) were cultured in 6-well plates (0.1 × 10^6^ cells/well) for 24 h in NC expansion medium (composition in Supplementary Material) and pre-treated with the FDA-approved drugs interleukin 1 receptor antagonist (IL-1Ra), growth and differentiation factor 5 (GDF-5), amiloride, metformin, and celecoxib at different concentrations (Table 1) for the last 3 h (21 h + 3 h). Afterwards, the drugs were removed and DDD mimicking condition (= hypoxia, low glucose, and medium supplemented with 1ow-grade pro-inflammatory cytokines TNFα, IL-1β, IL-6, all 100 pg/ml, full composition in Supplementary Material) was applied to the cells for further 24 h. Then, the cells were harvested and analyzed by reverse transcription quantitative PCR (RTqPCR) (chapter 4.3.1).

**TABLE 1:** FDA-approved drugs screened in nasal chondrocytes.

*Drug*	*Desired Effect*	*Concentrations*
Amiloride	Inhibitor of acid sensing ion channel 1/3 (Guo et al., 2019) which could prevent acid-induced decrease in cell proliferation and ECM gene expression (Liu et al., 2017)	10, 100 µM
Metformin	Inducer of inflammation resistant phenotype (Das et al., 2019) known for its chondroprotective properties (Park et al., 2019)	10, 100, 1000 µM
Celecoxib	Intradiscal delivery of celecoxib-loaded microspheres restored IVD integrity in preclinical canine model (Tellegen et al., 2018)	10, 100, 1000 µM
IL-1Ra	Inflammation inhibitor (Le Maitre et al., 2006) with matrix protective properties in intact human degenerate IVD explants (Le Maitre et al., 2007a)	10, 100, 500 ng/mL
GDF-5	Inducing NP-specific ECM forming effects (Le Maitre et al., 2009b) and driving differentiation of therapeutic cells towards NP-like cells (Colombier et al., 2016)	1, 10, 100 ng/mL

### 2.3 Generation of spheroids and nucleus pulposus microtissues

#### 2.3.1 Fabrication of nucleus pulposus and nasal chondrocyte spheroids

Nucleus pulposus spheroids (NPS) formation: 25′000 NP cells/well were seeded in 2% PolyHEMA (Sigma, P3932) coated 96-well plates and the formed spheroids were cultured for 14 days in NP differentiation medium (composition in Supplementary Material) in Thermo Scientific™ Heracell™ 150i CO_2_ incubator (37°C; 5% CO_2_, 20% O_2_). Media was changed twice a week. Nasal chondrocyte spheroids (NCS) formation: 12’500 NC/well were seeded in 2% PolyHEMA (Sigma, P3932) coated 96-well plates and the formed spheroids were cultured for 3 days in NC differentiation medium (composition in Supplementary Material) in Thermo Scientific™ Heracell™ 150i CO_2_ incubator (37°C; 5% CO_2_, 20% O_2_) without medium change.

#### 2.3.2 Preconditioning of nasal chondrocyte spheroids

NCS were formed in 96 well plates in NC differentiation medium (composition in Supplementary Material) for 3 days (Gryadunova et al., 2021) with/without GDF-5 (100 ng/mL), IL-1Ra (500 ng/mL) or the combination of both drugs. Then, the drugs were removed. Preconditioned NCS were either placed directly in DDD-mimicking condition (composition in Supplementary Material) for further 7 days and analyzed (chapter 4.3.2), or introduced into the NP microtissues (NPµT) model (see 4.3.3).

#### 2.3.3 Nucleus pulposus microtissue model

NPµT was cultured either alone or in combination with NCS or NC suspension. 16 NPS were pooled in a polypropylene conical tube to form the NPµT. 8 NPS and 16 NCS were pooled to form the NP µT + NCS. 8 NPS and 0.2 × 10^6^ NC cells were pooled to form the NPµT + NC cell suspension. The total number of the cells in each formed microtissues was 0.4 × 10^6^. The aggregates were cultured for 14 days in 0.5 mL of either healthy (normoxia, high glucose, NHG) or DDD-mimicking condition (= hypoxia, low glucose, and medium supplemented with 1ow-grade pro-inflammatory cytokines TNFα, IL-1β, IL-6, all 100 pg/mL, full composition in Supplementary Material). Hypoxia is native to both healthy and degenerated NP, so we used this condition to mimic DDD (Chen et al., 2014; Kwon et al., 2017). The medium was changed twice per week.

### 2.4 Histological, biochemical and molecular characterization

#### 2.4.1 Gene expression analysis

ECM genes (aggrecan, collagen type II) are downregulated in IVD degenerative condition (Le Maitre et al., 2007b) and inflammatory/catabolic genes (IL-6, IL-8, MMP3, MMP13) are upregulated (Goupille et al., 1998; Roberts et al., 2000; Le Maitre et al., 2007b; Teixeira et al., 2018). Thus, these targets were analyzed to verify whether pre-conditioning of NC (either in monolayer or 3D spheroidal organization) could modulate ECM degradation and inflammation. Total RNA from 0.2 M cells was extracted using the RNeasy Mini Kit (Quiagen, 74106), according to the manufacturer’s protocol. The RNA yield and purity were measured on a NanoDrop 1000 Spectrophotometer (Thermo Fisher Scientific, United States). SuperScriptTM III Reverse Transcriptase kit (Invitrogen, 18080093) was used to reverse-transcribe 0.5 μg of RNA into cDNA in a 30 μL volume. 10 ng of cDNA/well was mixed with TaqManTM Universal PCR Master Mix (Applied Biosystems, 4304437), RNase-free water, and TaqMan primers (ACAN: Hs00153936; COL2A1: Hs00264051; COL1A1: Hs00164004; MMP3: Hs00968305_m1; MMP13: Hs00233992_m1; IL-8: Hs00174103_m1; IL-6: Hs00985639_m1) in a total volume of 10 μL and used for quantitative real-time polymerase chain reaction performed on a 7300 Real-time PCR System (Applied Biosystems, United States). For each sample, Ct values of the target were subtracted from the Ct values of a housekeeping gene (human GAPDH, Hs02758991, Applied Biosystems) to derive the ∆Ct. Gene expression was quantified relative GAPDH (2^−ΔΔCT^) and relative to control (2^−ΔΔCT^).

#### 2.4.2 Biochemical content quantification

NPµT (+NCS/NC suspension) were digested for 16 h at 56°C in 1 mg/mL proteinase K solution [1 mg/ml proteinase K (Sigma-Aldrich, P2308) in 50 mM Tris (Sigma-Aldrich, A5456-3) with 1 mM EDTA (Fluka, 03680), 1 mM iodoacetamide (Sigma-Aldrich, I-1149) and 10 mg/mL pepstatin A (Sigma-Aldrich, P5318)]. Glycosaminoglycan (GAG) content was determined spectrophotometrically using Blyscan GAG Assay (Biocolor, B1000). DNA content was measured using the CyQuant Cell Proliferation Assay Kit (Invitrogen, C7026), with bacteriophage λ DNA as a standard, according to the manufacturer’s protocol. Total collagen content was quantified using the Hydroxyproline (HYP) Assay Kit (Sigma-Aldrich, MAK008) according to manufacturer’s protocol. Both GAG and HYP contents were normalized either to single NCS or to DNA content.

#### 2.4.3 Enzyme-linked immunosorbent assay (ELISA) and luminex

The amounts of MMP13, IL-8, and IL-1Ra in the cell culture media were quantified using ELISA. MMP13 and IL-8 are known to be released in IVD tissues experiencing catabolic shift (Le Maitre et al., 2004; Krock et al., 2019). SensoLyte Plus™ 520 MMP13 Assay Kit (Catalog #: 72019) was used for fluorometric detection of total MMP13, performed according to the manufacturer’s protocol. Human IL-8 ELISA Set (555244, BD) with ELISA reagent set B (550534, BD) were used to detect IL-8 according to the manufacturer’s instructions. IL-1Ra was quantified using the Human Luminex Discovery Assay (Magnetic Luminex Assay 2 Plex, bio-techne, LXSAHM-02) according to the manifacturer’s instructions.

#### 2.4.4 Histology and immunohistochemistry

Samples were fixed in 4% paraformaldehyde (01-1,000, formafix), washed in phosphate buffered saline (PBS), embedded in Richard-Allan Scientific HistoGel™ (HG-4000-012, ThermoFisher) and processed using Tissue Processing Center TPC 15 Duo (Medite, Germany). 4 μm-thick sections were cut (Microm HM 430 or Microm HM 340E) and collected on poly-L-lysine coated glass slides (J2800AMNZ, Fishersci). After dehydration, safranin-O/fast green (SafO/FG; SafO: 84120, Sigma; FG: F-7252, Sigma) stain with hematoxylin (J.T. Baker, MFCD00078111) nuclear counterstaining was performed to visualize the PG within the sections. Widefield microscopy (Nikon Ti2, Japan; acquisition software: Nikon NIS; Camera: Nikon DS-Ri2; Objective: ×20 or 40x; NA: 0.95) was applied for imaging. Images were processed using Fiji/ImageJ software (NIH, Bethesda, MD). For immunohistochemistry, sections were subjected to enzymatic epitope retrieval and blocked with 1% bovine serum albumin (BSA) (A9647, Sigma) supplemented with triton X-100 (1:1,000, 93418, Sigma), followed by application of primary antibodies anti-cleaved caspase 3 (1:300, polyclonal, 9661, Cell Signalling) and anti-GFP (1:1,500, GFP-1020, Aves). Respective matching secondary antibodies Alexa Fluor 647- or 488-conjugated (1:500, polyclonal, A21245, Invitrogen and 103–605-155, Jackson) were used, with DAPI as a nuclear counterstain. Widefield fluorescence microscopy (Nikon Ti2, Japan; acquisition software: Nikon NIS; Camera: Photometrics Prime 95B; Objective: 20 or 40x; NA: 0.95) was applied for imaging. Images were processed using Fiji/ImageJ software (NIH, Bethesda, MD).

#### 2.4.5 Image quantification

**FIGURE 2:**
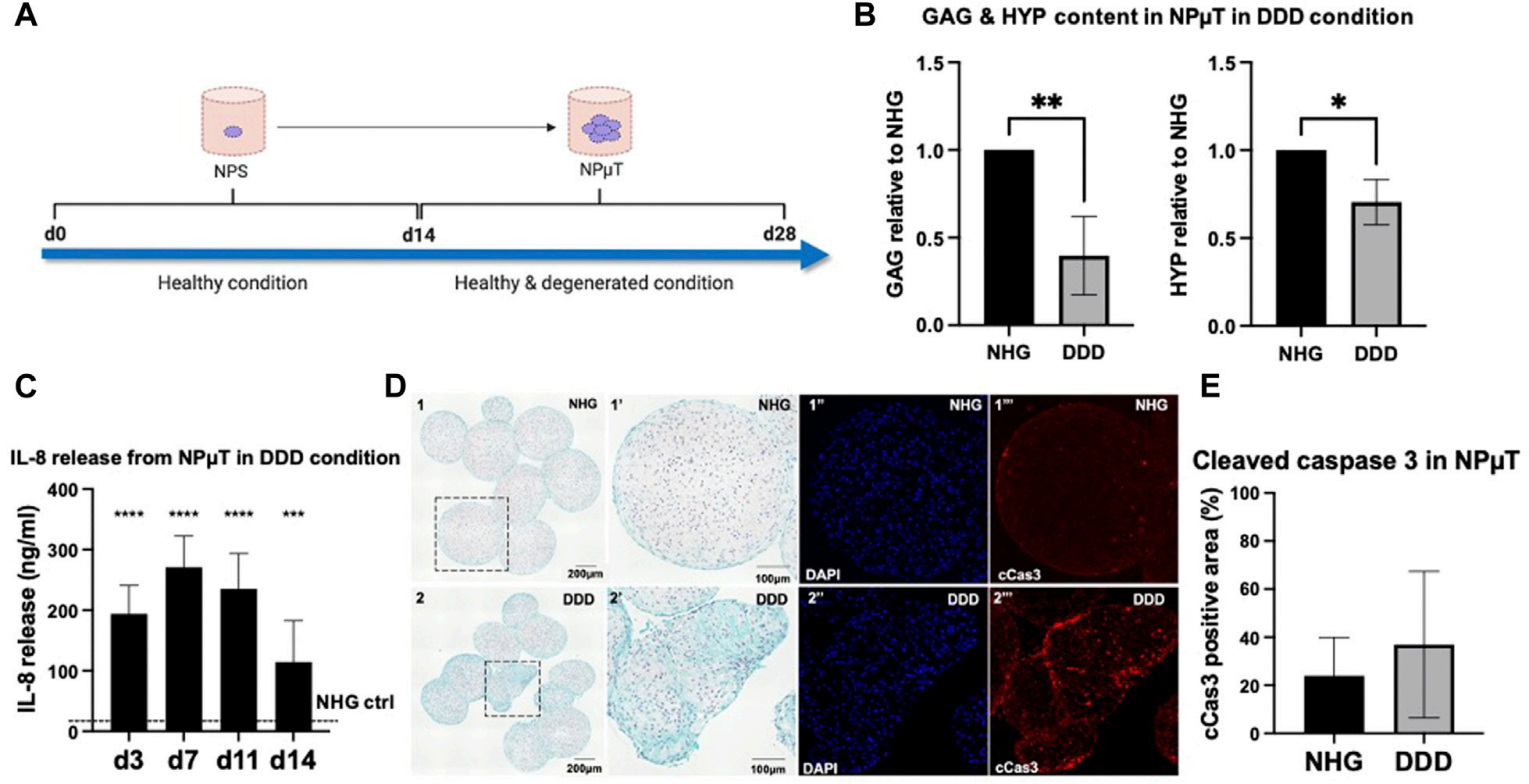
Development of 3D degenerative NP microtissue (µT) model. **(A)** NPµT was formed for 14d in NHG or DDD condition using NP spheroids (NPS) pre-cultured in healthy (NHG) condition for 14d. After 14d in NHG or DDD condition, NPµT was analyzed **(B)** biochemically by quantifying glycosaminoglycans (GAG) and collagens (HYP) (*n* = 6, mean ± SD, *p < 0.05, ANOVA). **(C)** Catabolic shift in degenerative NPµT was assessed by measuring IL-8 release in culture medium on day 3, 7, 11 and 14. Dashed line represents IL-8 release in NHG control (no IL-8 detected) (*n* = 5, mean ± SD, *p < 0.05 vs. NHG ctrl., ANOVA). (**D**) NPµT formed in (**D1**) NHG or (**D2**) DDD condition was stained with SafO/FG (**(D1’’)** zoomed D1 showing healthy cells producing proteoglycans, **(D2’’)** zoomed D2 showing cells with moderate proteoglycan production) and immunofluorescence (**(D1’’**-**2’’)** DAPI and **(D1’’’-D2’’’)** cleaved caspase 3 (cCas3) visualising nuclei (in blue) and apoptotic cells (in red), respectively) (*n* =6). **(E)** Quantification of cCas3 staining in NPµT (*n* = 6, mean ± SD, *p < 0.05, ANOVA).

**FIGURE 3:**
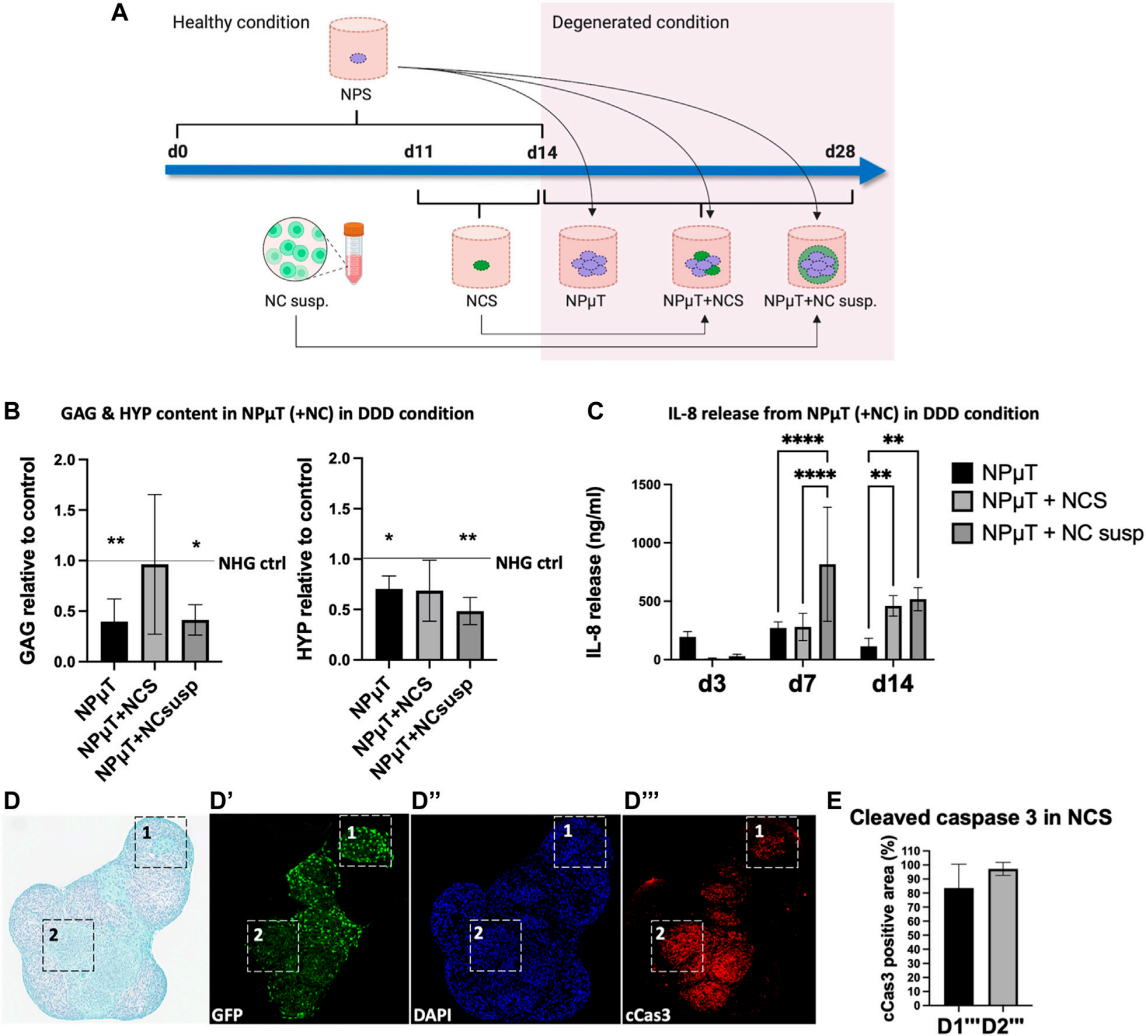
Assessing the responses of NC in 3D in vitro degenerative NPµT model. **(A)** NP spheroids (NPS) were formed for 2 weeks and NC spheroids (NCS) were formed for 3 days in healthy condition. After pooling NPS with either NCS or NC cell suspension (NC susp.), the aggregates were cultured for 2 weeks in either healthy or degenerated condition. Afterwards, **(B)** Glycosaminoglycans (GAG) and hydroxyproline (HYP) were quantified. Dashed lines represent value of healthy control. Asteriks indicate significance compared to healthy control (NP: *n* = 6, NC: *n* = 3, mean ± SD, *p < 0.05 vs. NHG ctrl., ANOVA). **(C)** Catabolic shift was assessed by measuring IL-8 release on day 3, 7, and 14. No IL-8 release was detected in healthy condition (NP: *n* = 6, NC: *n* = 3, mean ± SD, *p < 0.05, ANOVA). **(D)** SafO/FG staining of NPµT containing NCS accumulating proteoglycans (D1) and (D2) less/no proteoglycans. Immunofluorescence staining visualising (D’) GFP transduced NC, (D’’) nuclei (DAPI), and (D’’’) apoptotic cells (cleaved caspase 3 (cCas3)). Black and white squares depict zoomed-in subsections of (D1’’’) less apoptotic and (D2’’’) more apoptotic NCS (NP: *n* = 1, NC: *n* = 3). **(E)** Semi-quantification of cCas3 staining in D1’’’ and D2’’’ (NP: *n* = 1, NC: *n* = 3, mean ± SD, *p < 0.05, Mann-Whitney U test).

Multiplexed fluorescence images from tissue sections were analyzed with QuPath version 0.3.0, an open source software for whole-slide images (Bankhead et al., 2017). Immunopositive areas containing NCS were used as regions of interest (ROIs). StarDist extension, a deep-learning-based model for nuclei detection, was applied to the DAPI channel of fluorescent images to calculate the amount of cells in ROI. For each image, the mean intensity value representing cleaved caspase 3 (cCas3) was obtained. The mean fluorescence intensity was normalized to either number of cell detections or the area. The percentage of cCas3-positive areas was assessed by adjusting software built-in pixel classifier. 29 images were analyzed for experimental group 1 (Figure 2E), 8 images for group 2 (Figure 3E), and 28 images for group 3 (Figure 6G).

#### 2.4.6 Cell viability assay

The cell viability of NCS cultured in DDD mimicking conditions was assessed using the CellTiter-Glo Luminescent Cell Viability Assay (Promega, G7570) on day 0, 3, and 7 according to the manufacturer’s protocol. Briefly, the microplate with NCS was left at room temperature (RT) for 30 min prior to examination. Four empty wells were filled with 100 µl of the corresponding medium to obtain a value of background control. CellTiter-Glo Reagent and medium (ratio 1:1) were added to each well and mixed to induce cell lysis. Afterwards, the plate was incubated for 1 hour at RT to stabilize the luminescent signal. Luminescence was recorded using SPARK Multimode-Microplate Reader (Tecan, Switzerland). Luminescence signal proportional to cellular ATP generation was expressed in relative light units (RLU) and normalized to control (DDD ctr) (chapter 4.3.2).

### 2.5 Statistical analysis

All data were analyzed using GraphPad Prism software ver. 8.0.1 (GraphPad Software, Inc., La Jolla, Ca) and reported as mean ± SD. The following tests were used to assess the statistical significance: for normally distributed data, analysis of variance (ANOVA) followed by Sidak’s *post hoc* test (group analysis); for non-normally distributed data obtained from semi-quantitative image analysis, a non-parametric Mann-Whitney *U* test. Numerical values of probability (p) smaller than 0.05 were considered as statistically significant.

## 3 Results

### 3.1 Development of 3D *in vitro* degenerative nucleus pulposus microtissue model

To recapitulate the conditions during early stage IVD degeneration such as the pro-inflammatory/catabolic shift, onset of ECM degradation and mild apoptosis (Dou et al., 2021), pre-formed spheroids consisting of NP cells (NPS) were pooled to form NPµT at NHG (control group) or DDD (early stage IVD degeneration group) for 14d (Figure 2A). This 2-stage NPµT formation allows for generation of larger microtissues with low risk of necrotic core formation. This configuration also permits straightforward incorporation of therapeutic cells into 3D NP microenvironment with cell-produced ECM and factors playing a key role in NP degeneration such as low nutrition, acidity, hypoxia and pro-inflammatory cytokines. Histological and quantitative analysis revealed that NPµT formed in DDD microenvironment accumulated significantly less GAG and collagens compared to healthy NPµT (Figure 2B, D). NP cells also experienced catabolic shift (as demonstrated by enhanced release of IL-8), which confirmed the presence of the harsh DDD microenvironment (Figure 2C). Even though apoptotic cells were detected in subsections of the NPµT cultured in DDD microenvironment (Figure 2D‴), semi-quantification of the cleaved caspase 3 staining on the whole section revealed no significant upregulation of apoptosis in NPµT cultured in DDD compared to control (Figure 2E).

### 3.2 Assessing the performance of nasal chondrocytes in NP microtissue model

The NPµT model was designed to study the long-term effects of DDD microenvironment on putative therapeutic cells. As promising cell type for NP repair, NC were implemented in the model (Vedicherla and Buckley, 2017; Gay et al., 2019; Gryadunova et al., 2021). To evaluate possible differences in responses to DDD microenvironment, NC were incorporated in the model either as cell suspension (NPµT + NC cell suspension) or spheroids (NPµT + NCS). To generate the model, NPS were pooled with NCS or NC suspension and cultured for 2 weeks either in healthy (NHG) control condition, consisting of normoxia (20% O_2_) and high glucose (4.5 mg/ml), or in the aforementioned degenerative (DDD) condition (Figure 3A). Accumulation of ECM components within degenerative NPµT co-cultures was compared to NHG control and between NCS and NC suspension groups (Figure 3B). GAG and collagen content in NPµT + NCS group did not significantly differ from healthy control, while GAG and collagen in NPµT + NC suspension group was significantly reduced. No significant differences in GAG and collagen between NPµT + NCS and NPµT + NC cell suspension were detected, although trends towards higher ECM content in NPµT + NCS were observed. Addition of NCS to degenerative NPµT tended to increase GAG content, compared to degenerative NPµT only. Catabolic shift was measured by the release of IL-8, typical for DDD (Krock et al., 2019). On day 3 a trend towards reduced release of IL-8 by the NPµT + NCS (9 ± 5 ng/mL) and NC cell suspension (29 ± 17 ng/mL) was detected compared to NPµT (194 ± 47 ng/mL) (Figure 3C). On day 7 the IL-8 released by NPµT + NCS was significantly lower (280 ± 117 ng/mL) compared to NPµT + NC suspension (816 ± 488 ng/mL) but no difference could be shown on day 14. In NHG control, no IL-8 release was detected at any time point. As the incorporation of NCS into degenerative NPµT tended to increase GAG content, further (immuno-) histological analysis of NPµT + NCS group was performed. The staining indicated that NCS within the DDD microenvironment could accumulate proteoglycans (Figure 3D1) however not all of them to the same extent (Figure 3D2). cCas3 quantification in NPµT + GFP-NCS showed high presence of cells with apoptotic traits within GAG-negative areas (Figures 3D’’’2, E). To a limited extent, NCS survived and accumulated ECM compared to ECM content in NCS before implementation in the NPµT model (Supplementary Figure S1).

The 3D *in vitro* degenerative NPµT model was developed and tested using promising therapeutic cell type (NC). NC could be distinguished from NP cells within the model, allowing to explore the responses of both cell types to DDD microenvironment as well as the fate of the therapeutic NC. Furthermore, the model could be used to assess two cell configurations (cell suspension and spheroids). Our data suggested that even if NCS in the degenerated NPµT acquired apoptotic traits, their overall performance in the DDD microenvironment was superior to the one of NC suspension. One possibility to improve NCS performance for clinical use, while keeping regulatory requirements feasible, is to use pre-conditioning with FDA approved drugs. To optimize NCS function within the DDD microenvironment, the drugs should equip NC with anti-inflammatory and anti-catabolic resistance and/or enhance their anabolic activity.

### 3.3 Preconditioning of nasal chondrocytes to optimize their function in the nucleus pulposus

Cell preconditioning using hypoxia, inflammatory mediators, pharmacological drugs and chemical agents has been investigated to improve cell function, survival, and therapeutic efficacy in IVD field (Noronha et al., 2019). In order to facilitate clinical translation, we selected pre-conditioning of therapeutic NC using FDA approved drugs with anti-inflammatory, anti-catabolic, and/or anabolic activities, namely GDF-5, IL-1Ra, metformin, amiloride, and celecoxib. GDF-5 injection was shown to increase ECM accumulation in the IVD in clinical settings and IL-1Ra was reported to reduce anti-inflammatory and anti-catabolic factor release *in vitro, ex vivo,* and *in vivo*, partly by inhibiting the p38 MAPK activity (Le Maitre et al., 2007a; Studer et al., 2007). Celecoxib was shown to aid in restoring IVD integrity (Tellegen et al., 2018), amiloride could prevent acid-induced decrease in cell proliferation and ECM gene expression (Liu et al., 2017), and metformin was used to induce inflammation resistant phenotypes (Das et al., 2019; Park et al., 2019). A 3-stage experiment was designed i) to identify compounds/concentrations active in NC (2D screening), ii) to study the effects of pre-conditioning on NCS cultured in DDD microenvironment (3D pre-conditioning), and finally iii) to evaluate the function of pre-conditioned NCS using the compounds selected in i&ii within the degenerative NPµT model.

#### 3.3.1 Effects of compounds on nasal chondrocytes (2D drug screening)

**FIGURE 4:**
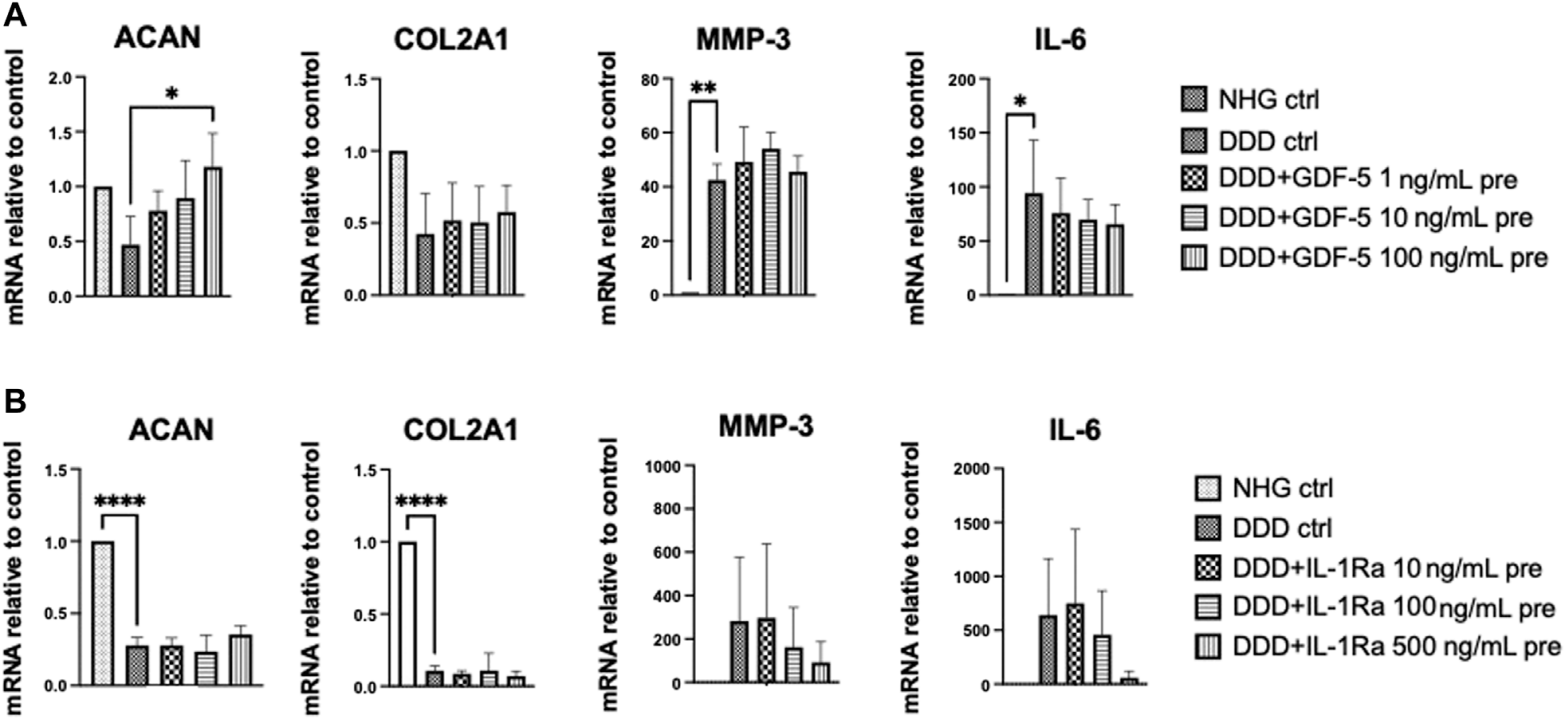
2D drug screening: Effects of drug pre-treatment on NC in DDD-mimicking conditions. Gene expression (relative to control) of anabolic genes (ACAN, COL2A1) and catabolic genes (MMP-3, IL-6) in NC pre-treated with **(A)** GDF-5 or **(B)** IL-1Ra. NHG: healthy condition, DDD: DDD mimicking condition (*n* = 3, mean ± SD, *p < 0.05, ANOVA).

NC were pre-treated with increasing concentrations of GDF-5, IL-1Ra, metformin, celecoxib, and amiloride for 3 h. Afterwards the drugs were removed, DDD mimicking condition was introduced to the cells for further 24 h, and the expression of anabolic (aggrecan: ACAN; collagen type II A1: COL2A1) and catabolic genes (matrix metallopeptidase 13: MMP-3; interleukin 6: IL-6) were analyzed. Metformin, amiloride and celecoxib pre-treated NC showed no significant modulation of tested genes (Supplementary Figure S2). GDF-5 (100 ng/mL) pre-treatment significantly upregulated ACAN and showed trend towards increased COL2A1 expression in the NC cultured in DDD mimicking condition (Figure 4A). In IL-1Ra (500 ng/mL) pre-treated NCS, a trend towards downregulated IL-6 and MMP-3 gene expression was detected (Figure 4B). Therefore, both GDF-5 (100 ng/mL) and IL-1Ra (500 ng/mL) were considered suitable for pre-conditioning of NCS.

#### 3.3.2 Preconditioning of nasal chondrospheres with IL-1Ra and GDF-5

NCS were pre-conditioned during their formation with IL-1Ra (500 ng/mL, putative anti-inflammatory activity), GDF-5 (100 ng/mL, putative anabolic activity), or the combination of both, and introduced to DDD mimicking conditions for 7 days. Pro-inflammatory/catabolic (IL-8, MMP-3) and anabolic (COL2A1, COL1A1, ACAN) gene expression, IL-8 release, GAG and total collagen content in the NCS were assessed on day 0 (before implementing in DDD condition), day 3 and 7. Furthermore, NCS viability was determined, with no significant difference between pre-treated NCS and DDD control.

##### 3.3.2.1 IL-1Ra pre-conditioning

**FIGURE 5:**
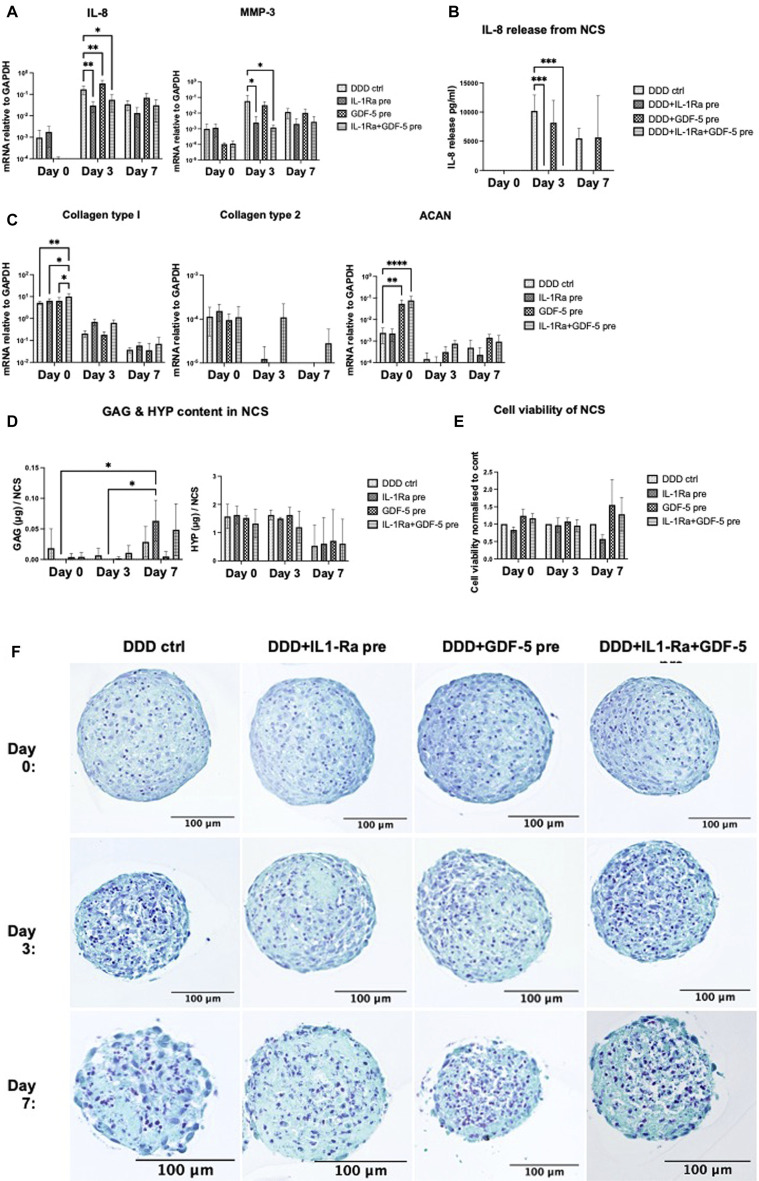
(Continued)

IL-1Ra preconditioning of NCS did not influence the expression of tested genes on day 0 (Figures 5A, C). In DDD condition, IL-1Ra pre-conditioned NCS significantly downregulated IL-8 and MMP-3 on day 3 and tended to reduce it on day 7 (Figure 5A). Pre-conditioning of NCS with IL-1Ra significantly downregulated the release of IL-8 protein on day 3 and on day 7 (trend), confirming gene expression data (Figure 5B). Although no significant effects on anabolic genes were observed (Figure 5C), IL-1Ra pre-conditioned NCS contained significantly more GAG (but not collagen) on day 7 compared to days 0 and 3 (Figure 5D), indicating that these NCS could accumulate ECM in DDD condition possibly *via* anti-catabolic action of IL-1Ra.

##### 3.3.2.2 GDF-5 pre-conditioning

Significant upregulation of ACAN expression (Figure 5C) and a trend towards downregulated IL-8 and MMP-3 expression (Figure 5A) were observed on day 0, before the GDF-5 pre-treated NCS were implemented in DDD condition. In DDD condition, GDF-5 pre-conditioning of NCS had no effect on the expression of tested genes (Figures 5A, C), nor IL-8 release (Figure 5B) or ECM accumulation (Figure 5D).

##### 3.3.2.3 IL-1Ra and GDF-5 pre-conditioning

At day 0, significant upregulation of COL1A1 and ACAN expression (Figure 4C) and reduced IL-8 and MMP-3 expression (not significant) were detected, likely due to the effects of GDF-5. In DDD condition, IL-1Ra + GDF-5 pre-conditioning significantly downregulated IL-8 and MMP3 genes in day 3 NCS (likely due to IL-1Ra) (Figure 4A) and tended to upregulate COL2A1 on days 3 and 7, possibly as a result of combination treatment (Figure 4C). The significant downregulation of IL-8 release from IL-1Ra + GDF-5 NCS on day 3 and on day 7 (trend) confirmed gene expression data, as expected effects of IL-1Ra (Figure 5B). Despite expectations, ECM accumulation in IL-1Ra + GDF-5 pre-conditioned NCS during 7 days was not significantly different from the corresponding controls (Figure 5E).

Altogether, GDF-5 pre-conditioning exerted some anabolic responses (i.e., increased ACAN expression). However, they were lost during NCS culture in DDD condition. IL-1Ra pre-conditioning downregulated pro-inflammatory (the expression of IL-8 on gene and protein level) and catabolic traits (reduced MMP-3 gene expression), which could allow for GAG accumulation in DDD condition (Risbud and Shapiro, 2014). Histological analysis also indicated superior effect on structural and cellular integrity when NCS was pre-conditioned with IL-1Ra (day 3 and 7) compared to control (Figure 5F). Since it was feasible to achieve anabolic effects in NCS without GDF-5 pre-conditioning, we proceeded to test the performance of IL-1Ra pre-conditioned NCS within the degenerative 3D *in vitro* NPµT model.

#### 3.3.3 Performance of NCS pre-conditioned with IL-1Ra within degenerative NP microtissue model

**FIGURE 6:**
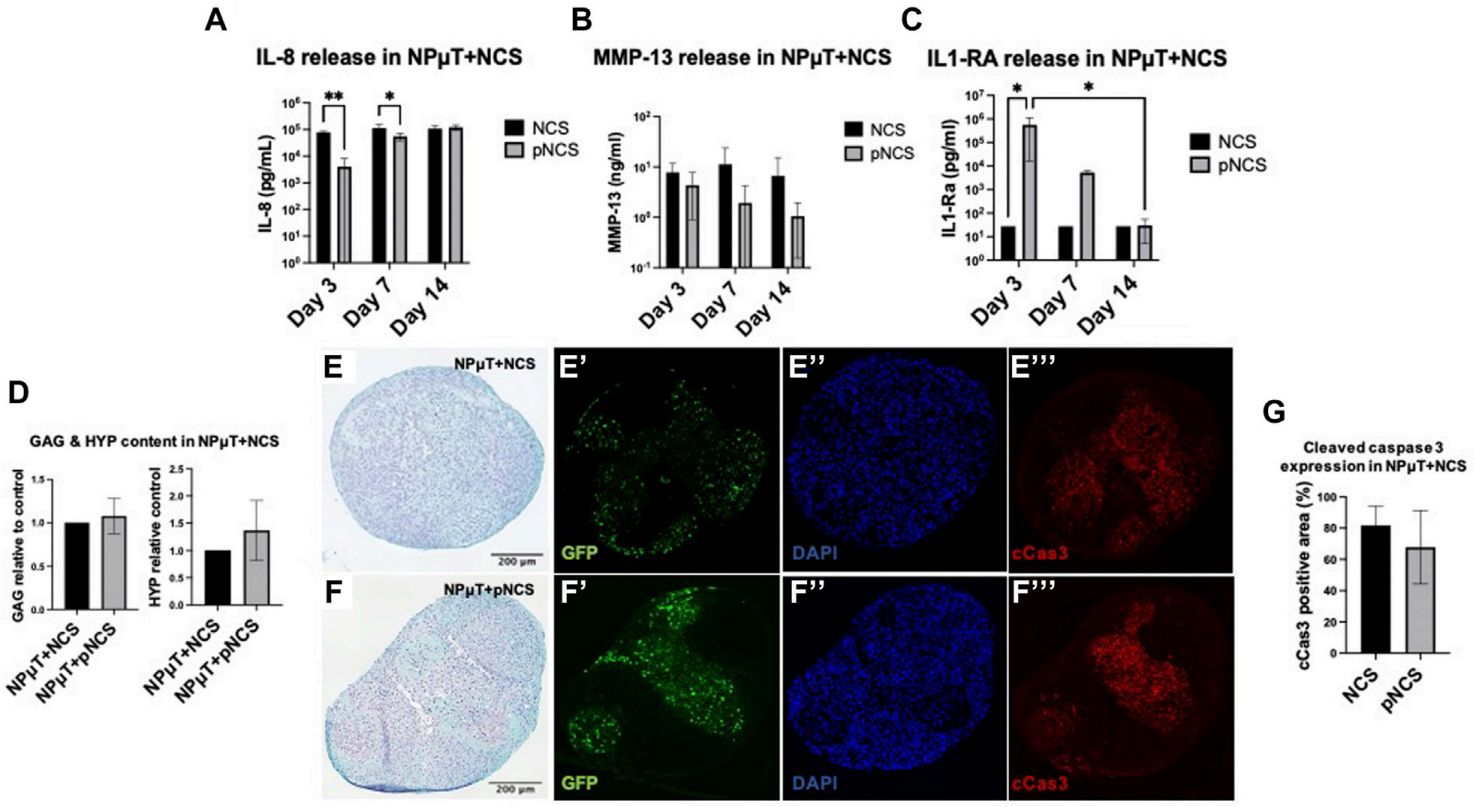
Implementation of pre-conditioned NCS (pNCS) in NPµT model. **(A)** IL-1Ra, **(B)** MMP-13, **(C)** IL-1Ra release from NPµT + NCS/pNCS was assessed on day 3, 7, and 14 (NP: *n* = 1, NC: *n* = 3, mean ± SD, *p < 0.05, ANOVA). **(D)** Glycosaminoglycan (GAG) and hydroxyproline (HYP) quantification, normalised to NPµT + NCS (NP: *n* = 1, NC: *n* = 3, mean ± SD, *p < 0.05, ANOVA). SafO/FG staining of NPµT containing **(E)** NCS and **(F)** pNCS, visualising proteoglycans. Immunofluorescence staining visualising (**E’&F’**) GFP transduced NC, (**E’’&F’’**) nuclei (DAPI), and (**E’’’&F’’’**) apoptotic cells (cleaved caspase 3, cCas3) within NPµT + NCS/pNCS. **(G)** Quantification of cCas3 staining (NP: *n* = 1, NC: *n* = 3, mean ± SD, *p < 0.05, ANOVA).

NCS or IL-1Ra pre-conditioned NCS (pNCS) were implemented in the NPµT model and cultured for 2 weeks in DDD microenvironment. Before implementation into the microtissue model, ECM content in NCS and pNCS was comparable (Supplementary Figure S3). Interestingly, throughout their co-culture in the NPµT model, pNCS released IL-1Ra up to day 7, possibly as a result of its entrapment in newly generated ECM of pNCS (Martino et al., 2014; Tan et al., 2021) (Figure 6C). During co-culture in DDD condition, the NPµT + pNCS released significantly less IL-8 on day 3 and 7 compared to NPµT + NCS without pre-conditioning (Figure 6A). Similar trend was observed for MMP-13 release (Figure 6B). However, on day 14, the amount of released IL-8 became comparable between NPµT + pNCS and NPµT + NCS, which could be explained by significant decrease of IL-1Ra release from pNCS at this timepoint (Figure 6C).

No significant difference in GAG and total collagen content was detected between NPµT + pNCS and NPµT + NCS (Figure 6D) after 2 weeks culture in DDD condition. Consistently, no difference in GAG content could be observed histologically (Figures 6E, F). Regarding NCS viability, no difference in cCas3 staining intensity could be visualized (Figures 6E’’’, F’’’). However, a trend towards reduced apoptosis within NPµT containing pNCS could be seen when the images were semi-quantified (Figure 6G).

Although the biological half-life of IL-1Ra protein has been reported to be 4–6 h (Akash et al., 2013), NPµT co-cultured with IL-1Ra pre-conditioned NCS exhibited superior anti-inflammatory/anti-catabolic properties, compared to NPµT with non-preconditioned NCS. However, IL-1Ra pre-conditioning was not sufficient to significantly increase the ECM content in NPµT, which could have been the consequence of reduced IL-1Ra release/activity in time, together with the presence of inflamed NP cells.

## 4 Discussion

We developed a new 3D *in vitro* degenerative NPµT model allowing to investigate the responses of therapeutic cells (in this case NC) to DDD microenvironment. Our NPµT model contains hypoxia, acidity, low-grade inflammation as well as 3D degenerated human NP cells with cell-made matrix, pre-stimulated with DDD condition. This model exhibits important features of early-stage IVD degeneration including degrading ECM and catabolic shift (Buckley et al., 2018; Ju et al., 2020). Therapeutic cells can be incorporated into the NPµT model either as cell suspensions or spheroids, acquiring/maintaining 3D organization of target NP tissue. In this model, NC cell suspension showed inferior GAG and collagen accumulation and an increased catabolic shift compared to NCS, supporting the use of NCS for clinical IVD repair. As spheroidal organization did not completely recover early-stage DDD signs, we attempted to improve the performance of NCS by drug pre-conditioning during their formation. From five tested candidates (amiloride, celecoxib, IL-1Ra, metformin, GDF-5), a clinically available anti-inflammatory drug IL-1Ra was evaluated as the most promising. Pre-conditioning of NCS with IL-1Ra further downregulated pro-inflammatory and catabolic responses, which could allow for GAG accumulation in DDD-mimicking condition. Moreover, pre-conditioned NCS exhibited long-term IL-1Ra release associated with reduced IL-8 and MMP13, which underlines the importance of anti-inflammatory pre-conditioning for IVD repair.

In patients, early stage DDD could still be restored using cell therapy (Buckley et al., 2018). An ideal *in vitro* model of early-stage DDD should accurately simulate the target tissue, by using NP cells from a patient with an appropriate Pfirrmann grade (Schwarzer et al., 1995; Luoma et al., 2000), as well as degenerative microenvironmental cues such as low glucose and oxygen levels, pH, and low-grade inflammation (Buckley et al., 2018). 3D alginate beads and pellet cultures are commonly used in IVD research to study the interaction of NP cells with a therapeutic cell source of interest *in vitro* (Gantenbein-Ritter and Chan, 2012; Ouyang et al., 2017). In alginate bead co-cultures, expanded NP and cells of interest are mixed and then differentiated together within the beads, while the alginate represents an exogenous ECM (Arkesteijn et al., 2015; Naqvi and Buckley, 2015). Alternatively, a transwell system is used for indirect co-cultures, to study paracrine interactions between both cell types encapsulated within alginate beads separately (Stoyanov et al., 2011; Song et al., 2015). The pellet culture model overcomes several disadvantages of alginate beads such as lack of reproducibility and uniformity of quality/size of the microspheres (Lee et al., 2001). The pellet culture system was extensively used for direct co-culture studies, where the therapeutic cells were mixed together with the expanded NP cells and centrifuged in a tube to form a pellet, thus differentiated together thereafter (Vadala et al., 2008; Chen et al., 2009; Svanvik et al., 2010). However, the simple pellet culture model does not accurately mimic the early stage *in vivo* DDD, thus is not ideal for investigating/testing cell therapies.

In clinical settings, the therapeutic cells are introduced to an already mature degenerative NP tissue containing differentiated NP cells and NP cell-produced matrix. Furthermore, the therapeutic cells create initial contacts with the ECM of the NP tissue rather than directly with the NP cells (Thorpe et al., 2018). Therefore, in order to mimic this situation *in vitro*, we designed new 3D degenerative NPµT model. To simulate clinical early-stage DDD, we first re-differentiated NP cells in spheroidal organization and allowed them to accumulate cell-produced ECM, to which the therapeutic cells could be introduced at later stage. We also stimulated the resulting NPS with hypoxia, low glucose, acidity, and low-grade inflammation, to recapitulate the chemical properties of degenerated NP (Buckley et al., 2018). Pooling the NPS with the therapeutic cells allows to create NP niches where the cells of interest could integrate into. Furthermore, the NPµT model also contains low nutrition, acidity, hypoxia and low-grade inflammation, thus can be used to study the long-term effect of degenerative NP microenvironment on therapeutic cells. As such, this straightforward yet sufficiently complex NPµT model overcomes limitations of currently used models.

For early stage IVD repair, therapeutic cells have to reside, survive, resist inflammation and produce ECM within the degenerative NP tissue. In previous studies, NC showed superior viability in simulated DDD microenvironment over commonly used MSCs and AC, thus represent a promising cell source for IVD repair (Vedicherla and Buckley, 2017; Gay et al., 2019; Borrelli and Buckley, 2020; Gryadunova et al., 2021). In degenerative IVD condition, NC were reported to produce a ratio of low collagen to high GAG content whereas AC produce less favorable high collagen ratios (Vedicherla and Buckley, 2017). We have previously demonstrated the potential of NC as spheroids (NCS) for IVD repair (Gryadunova et al., 2021). However, these studies were performed partially in absence of the NP cells, which could modulate the behavior of NC, or only the short-term DDD mimicking conditions were applied (Gay et al., 2019; Gryadunova et al., 2021). In the current study we implemented NC either as NCS or NC suspension in the degenerative NPµT model. In the NPµT, NCS survived and produced proteoglycans only partially, possibly due to the inflamed microenvironment which is known to affect cell viability and ECM degradation (Wang et al., 2020).

We have also shown that pre-conditioning of NCS is necessary to improve their ability to counteract inflammation, increase cell survival, and/or accumulate ECM. 2D drug screening revealed two promising candidates, IL-1Ra (anti-inflammatory, (Le Maitre et al., 2006; Le Maitre et al., 2007a)) and GDF-5 (anabolic, (Le Maitre et al., 2009b; Colombier et al., 2016)), with GDF-5 being eliminated at next stage (3D) due to its inferior effects in NCS pre-conditioning tests. We expected anabolic effect of GDF-5 on NCS, as benefits of GDF-5 in cartilage and IVD repair are well described (Li et al., 2004; Wang et al., 2004; Chujo et al., 2006; Cui et al., 2008; Liang et al., 2010; Guo et al., 2021; Sun et al., 2021). IL-1Ra preconditioned NCS were further implemented into the NPµT model. Overall IL-1Ra preconditioning inhibited pro-inflammatory and catabolic responses of NCS (IL-8, MMP-3, MMP-13), which suggested IL-1Ra interference with MAPK/ERK and NFκB signaling pathways *via* binding to the IL-1 receptor, consistent with literature (Le Maitre et al., 2006; Le Maitre et al., 2007a; Risbud and Shapiro, 2014). While IL-1Ra promoted NCS GAG accumulation in DDD mimicking condition, it failed to produce similar significant effects in NPµT model. This result indicates that degenerated NP microtissue indeed influences the performance of therapeutic cells (compared to only chemical DDD-mimicking condition), thus NP microtissue should be present *in vitro* during preclinical therapeutic testing.

The biological half-life of IL-1Ra protein has been reported to be 4–6 h (Akash et al., 2013) which is too short for clinical IVD repair. Several approaches have been taken to prolong the half-life of IL-1Ra such as fusing IL-1Ra with proteins (elastin-like polypeptides, human serum albumin, albumin domain antibodies) or by combining it with biodegradable polymers (poly (D,L-lactidide-co-glycolide), PLGA, polyethylene glycol (PEG), thermo-reversible gel) to prolong its steady-state sustained release at the site of administration (Akash et al., 2013). However, another approach could be to entrap IL-1Ra within the ECM of the spheroids by pre-conditioning NCS during their formation time, as in our study. The entrapped IL-1Ra could be slowly released (Joshi et al., 2018), supporting cells to counteract inflammation for longer time periods. In our NPµT model, the anti-inflammatory protection by IL-1Ra lasted up to 14 days and appeared to inversely correlate with the release of IL-8, suggesting that the IL-1Ra entrapped within the NCS was consumed.

Currently our NPµT model has several limitations that should be addressed in the future. We used a ratio of 1:1 ratio for NP/NC cells based on literature (Strassburg et al., 2010; Tao et al., 2013; Dai et al., 2014; Ouyang et al., 2017). However, it was also reported that a ratio of 75:25 NP/MSC cells leads to optimized MSC differentiation towards NP phenotype (Richardson et al., 2006), thus it still has to be determined which ratio is optimal for co-culture studies of NP cells with therapeutic cells in NPµT model. Another limitation is that our microtissues were cultured in static conditions. Applying compressive loading to microtissues might even better mimic early stage IVD degeneration and increase cell survival due to enhanced nutrient diffusion and waste removal within the NPµT model.

The 3D *in vitro* degenerative NPµT model aims to substitute the use of current alginate and pellet culture systems for preclinical *in vitro* investigations of IVD cell therapies. The model allows to study the survival and performance of (primed) therapeutic cells within the NP microenvironment mimicking early stage IVD degeneration. An orthotopic animal model will be required to compare the function of therapeutic cells in the NPµT model vs. *in vivo*. As long-term follow-up, a sheep model will be used to evaluate whether IL-1Ra preconditioning of NCS would provide durable IVD repair or NCS will have to be combined with other strategies, e.g. enabling further increase of NCS anabolic activity.

## 5 Conclusion

Currently, there is no standardized 3D *in vitro* NP model available to study the responses of therapeutic cells to DDD microenvironment. In this study we developed a 3D model which includes differentiated NP cells, cell-produced matrix, and environmental cues associated with DDD microenvironment. By implementing NC within the model, we showed that the NC in spheroidal organization are superior to NC in suspension and have potential to survive and accumulate ECM components within DDD microenvironment. Furthermore, we provided evidence after testing five FDA approved drugs that IL-1Ra pre-conditioning of NCS provides anti-inflammatory and anti-catabolic effects. In future studies, the survival and function of IL-1Ra pre-conditioned NCS will be investigated within *ex vivo* bovine IVD explants cultured under loading and in an animal model.

## Data Availability

The raw data supporting the conclusion of this article will be made available by the authors, without undue reservation.
