# Supplementary material for: Human 3D nucleus pulposus microtissue model to evaluate the potential of pre-conditioned nasal chondrocytes for the repair of degenerated intervertebral disc
Source: Front Bioeng Biotechnol. 2023 Feb 14;11:1119009. doi: 10.3389/fbioe.2023.1119009 (PMC9971624; doi:10.3389/fbioe.2023.1119009)
Supplement: Supplementary file 1 [file DataSheet1.DOCX]

**Human 3D nucleus pulposus micro-tissue model to evaluate the potential of pre-conditioned nasal chondrocytes for the repair of degenerated intervertebral disc**

**Supplementary Material**

## Media composition

### Expansion Medium

#### NC Expansion Medium

Complete Medium (10% FBS): Dulbecco’s Modified Eagle Medium (DMEM) (Invitrogen, 10938-025) supplemented with 10% fetal bovine serum (FBS) (Invitrogen, 10270-106), 1mM sodium pyruvate (SP) (Invitrogen, 11360-039), 10mM 4-(2-hydroxylethyl)-1-piperazineethanesulfonic acid (HEPES) (Invitrogen, 16630-056), 100 units/ml penicillin, 100µg/ml streptomycin, 0.29 mg/ml L-glutamine (PSG) (Invitrogen, 10378-016), 10ng/ml transforming growth factor (TGF-β1) (R&D, 240-BO-10) and 5 ng/ml fibroblast growth factor (FGF2) (R&D, 233-FB-025).

#### NP Expansion Medium

Complete Medium F12 (10% FBS): Dulbecco’s Modified Eagle Medium Nutrient Mixture F-12 (DMEMF/12) (Invitrogen, 10938-025) supplemented with 10% FBS (Invitrogen, 10270-106), 1mM SP (Invitrogen, 11360-039), 10mM HEPES (Invitrogen, 16630-056), 100 units/ml penicillin, 100µg/ml streptomycin, 0.29 mg/ml L-glutamine (PSG) (Invitrogen, 10378-016) and 5 ng/ml FGF2 (R&D, 233-FB-025).

### Differentiation Medium

#### NC Differentiation Medium

NC chondrogeic medium: DMEM (31885-023, Gibco), 1 mM SP (Invitrogen, 11360-039), 10mM HEPES (Invitrogen, 15630-056), 100 units/ml penicillin, 100 μg/ml streptomycin, 0.29 mg/ml L-glutamine (PSG) (Invitrogen, 10378-016), 10 μg/ml Insulin-Transferrin-Selenium (Gibco, 51300-044), 0.56 mg Linoleic acid (Sigma, L9530-5), 1.25 ml/l human serum albumin (CSL Behring, 43075), 10ng/ml TGF-β3 (100-36E, Peprotech), 0.1μM dexamethasone (Sigma, D-2915) and 0.1mM ascorbic acid (Sigma, A-8960).

#### NP Differentiation Medium

NP chondrogenic medium: DMEMF/12 (Invitrogen, 10938-025) supplemented with 1 mM SP (Invitrogen, 11360-039), 10mM HEPES (Invitrogen, 15630-056), 100 units/ml penicillin, 100 μg/ml streptomycin, 0.29 mg/ml L-glutamine (PSG) (Invitrogen, 10378-016), 10 μg/ml Insulin-Transferrin-Selenium (Gibco, 51300-044), 0.56 mg Linoleic acid (Sigma, L9530-5), 1.25 ml/l human serum albumin (CSL Behring, 43075), 10ng/ml TGF-β3 (100-36E, Peprotech), 0.1μM dexamethasone (Sigma, D-2915) and 0.1mM ascorbic acid (Sigma, A-8960).

#### Normoxic High Glucose medium (NHG)

Dulbecco’s Modified Eagle Medium (Invitrogen, 10938-025) supplemented with 1mM SP, 10mM HEPES, 100 units/ml penicillin, 100 µg/ml streptomycin, 0.29 mg/ml L-glutamine (PSG) (Invitrogen, 10378-016), 10 µg/ml Insulin-Transferin-Selenium (Gibco, 51300-044), 0.56 mg/5.6µl Linoleic acid (Sigma, L9530-5), 1.25 ml/l human serum albumin (CSL Behring, 43075), 10ng/ml TGF-β3, 0.1µM dexamethasone (Sigma, D-2915) and 0.1mM ascorbic acid (Sigma, A-8960).

#### DDD mimicking medium (DDD)

For first 3 days: DMEM low glucose (Gibco, 11885-084) supplemented with 1mM SP, 10mM HEPES, 100 units/ml penicillin, 100µg/ml streptomycin, 0.29 mg/ml L-glutamine (PSG) (Invitrogen, 10378-016), 10 µg/ml Insulin-Transferin-Selenium (Gibco, 51300-044), 5.6µg/mL Linoleic acid (Sigma, L9530-5), 1.25 ml/l human serum albumin (CSL Behring, 43075), 0.45 mg/ml lactic acid (MP Biomedicals, ICN19022805) and 100 pg/ml of TNFα, IL1β, IL6 (All Sigma-Aldrich, SRP3177, GF331, SRP3096),10ng/ml TGF-β3, 0.1µM dexamethasone (Sigma, D-2915) and 0.1mM ascorbic acid (Sigma, A-8960). For further 11 days: DMEM low glucose (Gibco, 11885-084) supplemented with 1mM SP, 10mM HEPES, 100 units/ml penicillin, 100µg/ml streptomycin, 0.29 mg/ml L-glutamine (PSG) (Invitrogen, 10378-016), 10 µg/ml Insulin-Transferin-Selenium (Gibco, 51300-044), 5.6µg/mL Linoleic acid (Sigma, L9530-5), 1.25 ml/l human serum albumin (CSL Behring, 43075), 0.45 mg/ml lactic acid (MP Biomedicals, ICN19022805) and 100 pg/ml of TNFα, IL1β, IL6 (All Sigma-Aldrich, SRP3177, GF331, SRP3096).

## Supplementary tables

***Table S1:*** *Donor demographics and characteristics of nasal chondrocytes source tissues.*

|  | **NC1** | **NC2** | **NC3** |
| --- | --- | --- | --- |
| **Age** | 52 | 25 | 53 |
| **Sex** | male | female | female |
| **Source** | Rhinoplasty | Rhinoplasty | Rhinoplasty |
|  | **NC4** | **NC5** | **NC6** |
| **Age** | 53 | 26 | 34 |
| **Sex** | male | male | male |
| **Source** | Septoplasty | Rhinoplasty | Rhinoplasty |

***Table S2:*** *Donor demographics and characteristics of nucleus pulposus source tissues.*

|  | **NP1** | **NP2** | **NP3** |
| --- | --- | --- | --- |
| **Age** | 72 | 53 | 46 |
| **Sex** | male | male | male |
| **Source** | L3/4  grade 3 | L4/5  grade 3 | L5-S1  grade 3 |
|  | **NP4** | **NP5** | **NP6** |
| **Age** | 47 | 43 | 79 |
| **Sex** | female | male | female |
| **Source** | L5-S1  grade 3 | L4/5  grade 2.5 | L3/4  grade 2.5 |

## Supplementary figure


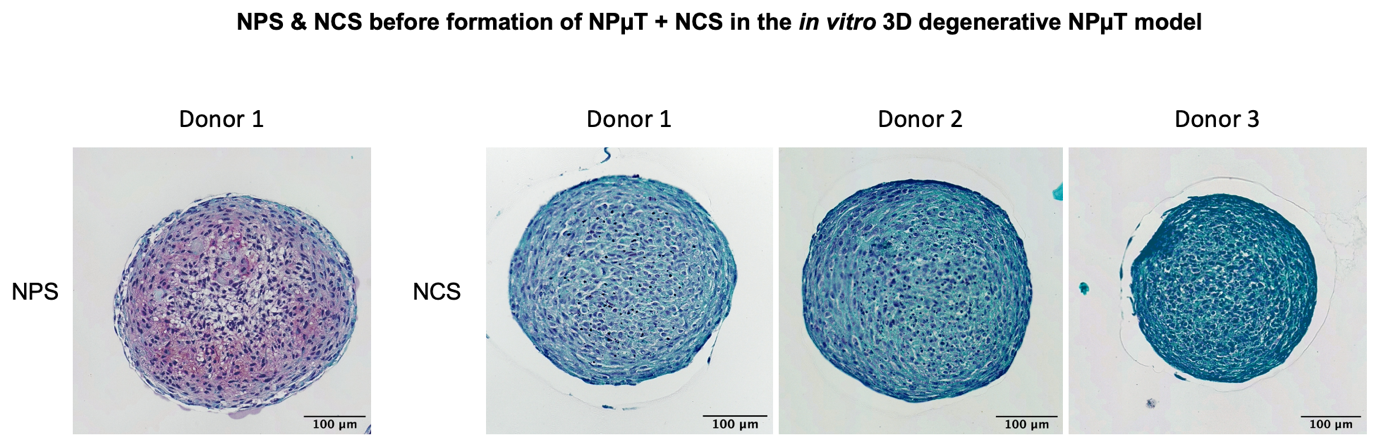


***Supplementary figure 1:*** *Safranin O staining of NPS & NCS before formation of NPµT + NCS in the in vitro 3D degenerative NPµT model.*

***Supplementary figure 2: Effects of drug pre-treatment on catabolic and anabolic marker expression in nasal chondrocytes (NC).*** *Relative gene expression (2^-dCt^) of anabolic markers (aggrecan, collagen type II) and catabolic markers (MMP3, IL-6) in amiloride, celecoxib, and metformin pre-treated NC. NHG: healthy condition, DDD: DDD mimicking condition. (n = 3, mean ± SD, *p < 0.05, ANOVA).*


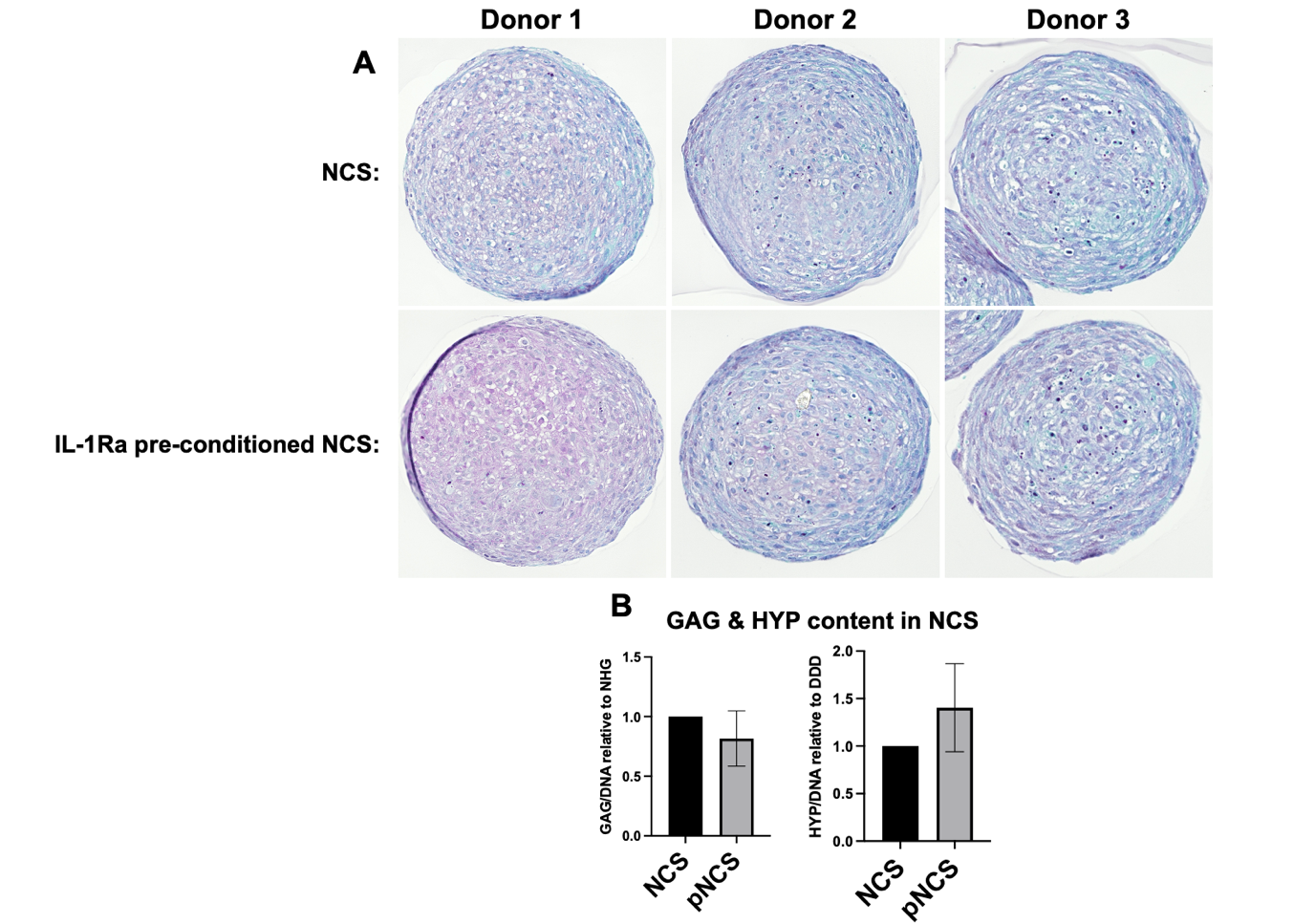


***Supplementary figure 3: NCS and IL-1Ra pre-conditioned NCS (pNCS) before implementation in the 3D in vitro NPµT model.*** *(A) SafO/FG staining of NCS and pNCS visualising proteoglycan content. (B) Proteoglycan and total collagen quantification in NCS and pNCS (n = 3, mean ± SD, *p < 0.05, ANOVA).*
